# A New Species of *Vietnamella* Tshernova 1972 (Ephemeroptera: Vietnamellidae) from Thailand

**DOI:** 10.3390/insects11090554

**Published:** 2020-08-20

**Authors:** Chonlakran Auychinda, Luke M. Jacobus, Michel Sartori, Boonsatien Boonsoong

**Affiliations:** 1 Animal Systematics and Ecology Speciality Research Unit (ASESRU), Department of Zoology, Faculty of Science, Kasetsart University, Bangkok 10900, Thailand; chonlakran.au@ku.th; 2 Division of Science, Indiana University Purdue University Columbus, 4601 Central Avenue, Columbus, IN 47203, USA; luke.jacobus@gmail.com; 3 Museum of Zoology, Palais de Rumine, Place Riponne 6, CH-1005 Lausanne, Switzerland; michel.sartori@vd.ch; 4 Department of Ecology and Evolution, Lausanne University, CH-1015 Lausanne, Switzerland

**Keywords:** COI, Ephemerelloidea, mayfly, integrative taxonomy

## Abstract

**Simple Summary:**

The monogeneric family Vietnamellidae is endemic in the Oriental region and found only in the Indomalayan biogeographic realm. Seven nominal species have previously been described, but three are considered to be junior synonyms. However, some provisional species designations have been established. In this study we described formally *Vietnamella* sp. C as a new species, *V. nanensis* sp. n., based on the specimens used for initial DNA data and our expanded set of materials from Nan Province, Thailand. Based on morphology, the larva of the new species can be distinguished from those of other congeners. Additional mitochondrial cytochrome oxidase subunit I gene data for the new species are also provided. A key to larvae of all known species in the genus is provided.

**Abstract:**

The larva, male subimago, female imago, and eggs of *V. nanensis* sp. n. are described based on specimens from Mae Hong Son and Nan provinces, Thailand. The female subimago is described based on a photograph of a specimen reared to the imago stage. The species previously was distinguished only by DNA barcode data and designated as *Vietnamella* sp. C. Based on morphology, the larva of the new species can be distinguished with the following combination of characteristics: (i) pattern of serration on the ventral margin of the forefemur, (ii) posterolateral margins of abdominal terga with pairs of acute tubercles, especially terga VI and VII, (iii) a well-developed pair of median ridge projections on tergum X, (iv) the second segment of the maxillary palp being about 1.3× the length of the third segment, and (v) females containing eggs with prominent protuberances on the chorionic surface. A key to larvae of all known species in the genus is provided.

## 1. Introduction

Mayflies (Ephemeroptera) are one of the most common components of benthic communities and contribute to ecosystem services [1,2,3,4,5]. They have been used as indicators of water quality [6,7,8,9]. Recently, the diversity of Thai mayflies is provided (e.g., Baetidae [10,11,12], Ephemerellidae [13], Heptageniidae [14,15,16], Leptophlebiidae [17,18], Prosopistomatidae [19], Teloganodidae [20], Tricorythidae [21]). Vietnamellidae is a monogeneric family of mayflies found only in the Indomalayan biogeographic realm. It may represent an extant basal lineage of the superfamily Ephemerelloidea, but more data are needed to test this hypothesis [22,23]. Seven nominal species have previously been described, but three are considered to be junior synonyms. All of the named species are known from Vietnam, China, and Thailand [24,25,26]. The species richness of the genus is greater, but lack of sufficient material and stage associations have hampered rapid progress in formally naming and describing this diversity. Until more materials and data are available, some provisional species designations have been established: *Vietnamella* sp. A from India [27], and *Vietnamella* sp. B & *Vietnamella* sp. C from Thailand [26]. Other material has been reported at the genus level from Laos, without provisional species designations [28].

From among the three provisional species, *Vietnamella* sp. C was only known from a few mitochondrial cytochrome c oxidase subunit I (COI) sequences that had been retrieved by Auychinda et al. [26] from the Barcode of Life Data System (BOLD). No previous attempt had been made to place the larval specimens from which the DNA had been obtained to a species, due to the poor state of systematics of the group (see, e.g., [25,29]). In light of the findings of Auychinda et al. [26], these specimens were investigated again, and new material was discovered, including the male subimago, female subimago, and egg stages. The present study aims to name and describe formally *Vietnamella* sp. C as a new species based on the specimens used for initial DNA data and our expanded set of materials from Nan Province, Thailand. Additional COI data for the new species are also provided, and these data were analyzed using Bayesian inference methods.

## 2. Materials and Methods

### 2.1. Sampling and Morphological Observations

The newly acquired specimens used for description and photography were collected from Nan Province in Northern Thailand. Larvae were collected from cobble in moderate to fast-flowing areas. Mature larvae were reared using earthenware pots connected to an air supply (Figure 8C) until emergence of winged stages. The specimens that have been used to obtain initial COI data were retrieved from L.M.J.’s research collection. The chorionic structure was investigated by drying the eggs, coating them with gold, and observing them with a FEI Quanta 450 Scanning Electron Microscope (SEM). The external structures were prepared on permanent slides using Euparal^®^ as a medium and observed by light microscopy. Final plates were prepared with Adobe Photoshop^®^ CC 2020. Holotype and paratype specimens of the new species are deposited in the collections of the Zoological Museum at Kasetsart University in Bangkok, Thailand [**ZMKU**] and the Museum of Zoology in Lausanne, Switzerland [**MZL**]. L.M.J.’s materials are currently stored in trays with other specimens used for DNA barcoding, and these units will be deposited in the Purdue University Entomological Research Collection, West Lafayette, Indiana, United States of America [**PERC**]. Our species hypotheses are based on the convergence of the morphological species concept and the phylogenetic species concept.

### 2.2. Molecular Analysis

The COI sequence of *Vietnamella nanensis* sp. n. was newly amplified from the Wa River, Nan Province, Thailand. The 658 bp sequence was included in the Bayesian tree reconstruction with the other vietnamellid mayflies from GenBank and the BOLD system. The preserved specimens were dissected (thorax) for DNA extraction. Total DNA was extracted using a genomic DNA purification kit (NucleoSpin, Macherey-Nagel, Germany) following the manufacturer’s protocol. The COI amplification was performed using LCO1490 and HCO2198 [30]. The polymerase chain reaction (PCR) conditions and procedure were performed as described previously [26]. Purification and sequencing were conducted by Macrogen, Inc. (Seoul, South Korea). The molecular protocols and Bayesian tree construction methods were as previously reported by Auychinda et al. [26]. Nucleotide sequences obtained in this study were deposited in the GenBank database. Other *Vietnamella* sequences were also obtained from the BOLD system and GenBank, and *Teloganella umbrata* Ulmer was used as an outgroup (a specimen of Narathiwat Province, Thailand, was amplified and sequenced by the authors); details are presented in Table 1. The Kimura 2-parameter (K2P) genetic distance was analyzed to confirm species delimitation.

### 2.3. Ethics Statement

The present study was approved by the ethics committee of Kasetsart University (approval no. ACKU61-SCI-029) for rearing and collecting the mayfly specimens.

## 3. Results

### 3.1. Taxonomy

**Figure 1:**
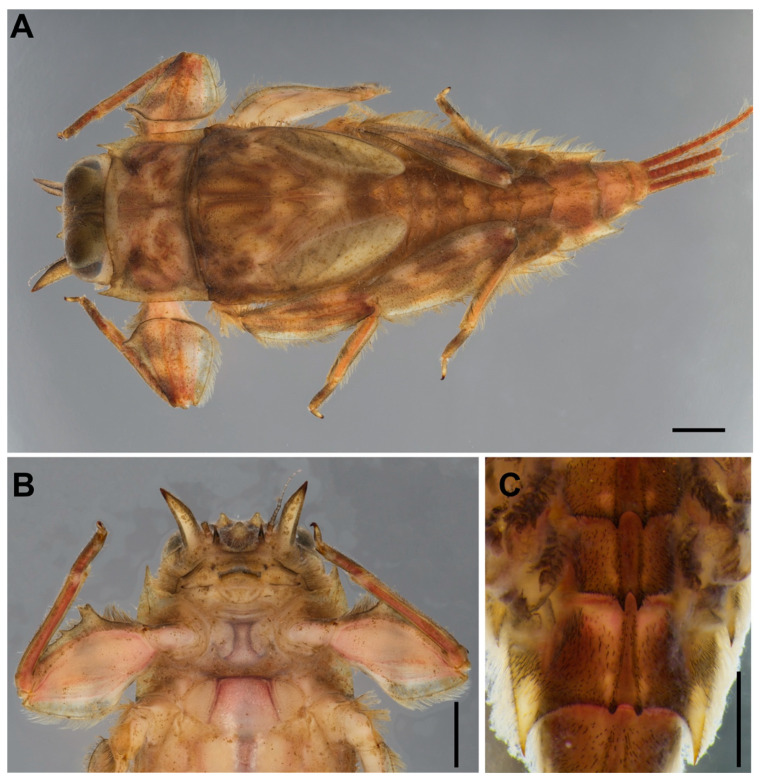
*Vietnamella nanensis* sp. n. (paratype) (**A**) habitus of larva; (**B**) ventral view of head and thorax; (**C**) abdominal terga VI–VII. Scale bars: 1 mm.

**Figure 2:**
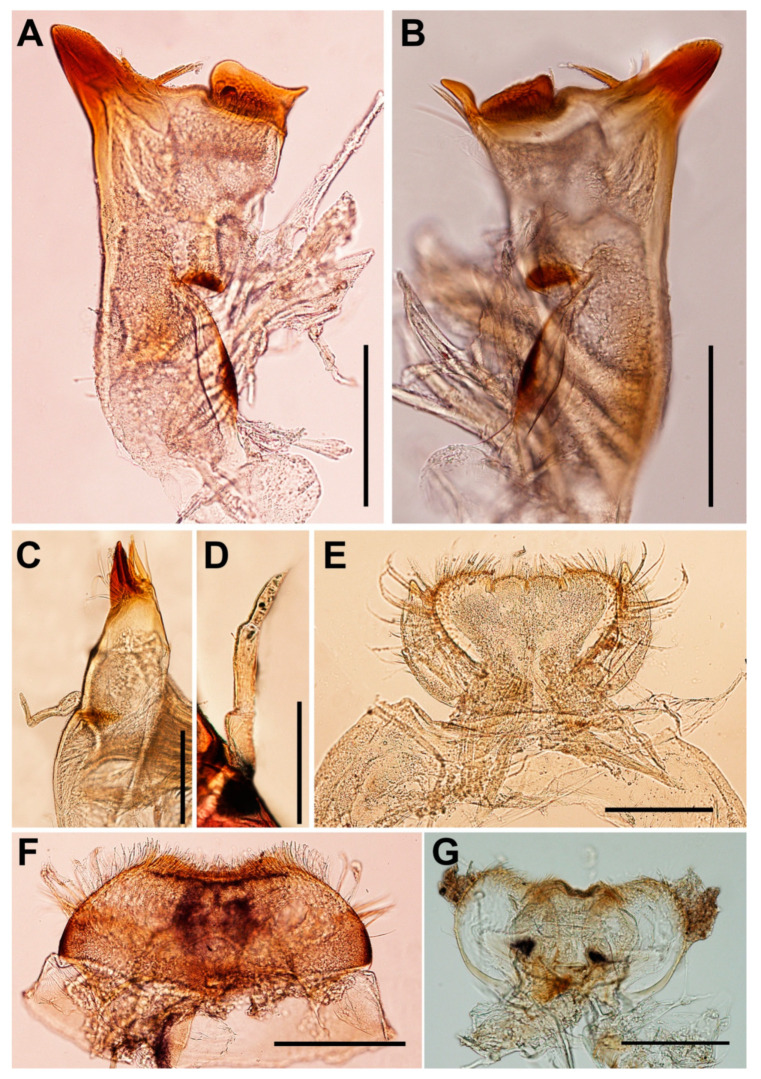
*Vietnamella nanensis* sp. n. (**A**) left mandible; (**B**) right mandible; (**C**) maxilla; (**D**) maxillary palp; (**E**) labium; (**F**) labrum; (**G**) hypopharynx. Scale bars: 0.2 mm.

**Figure 3:**
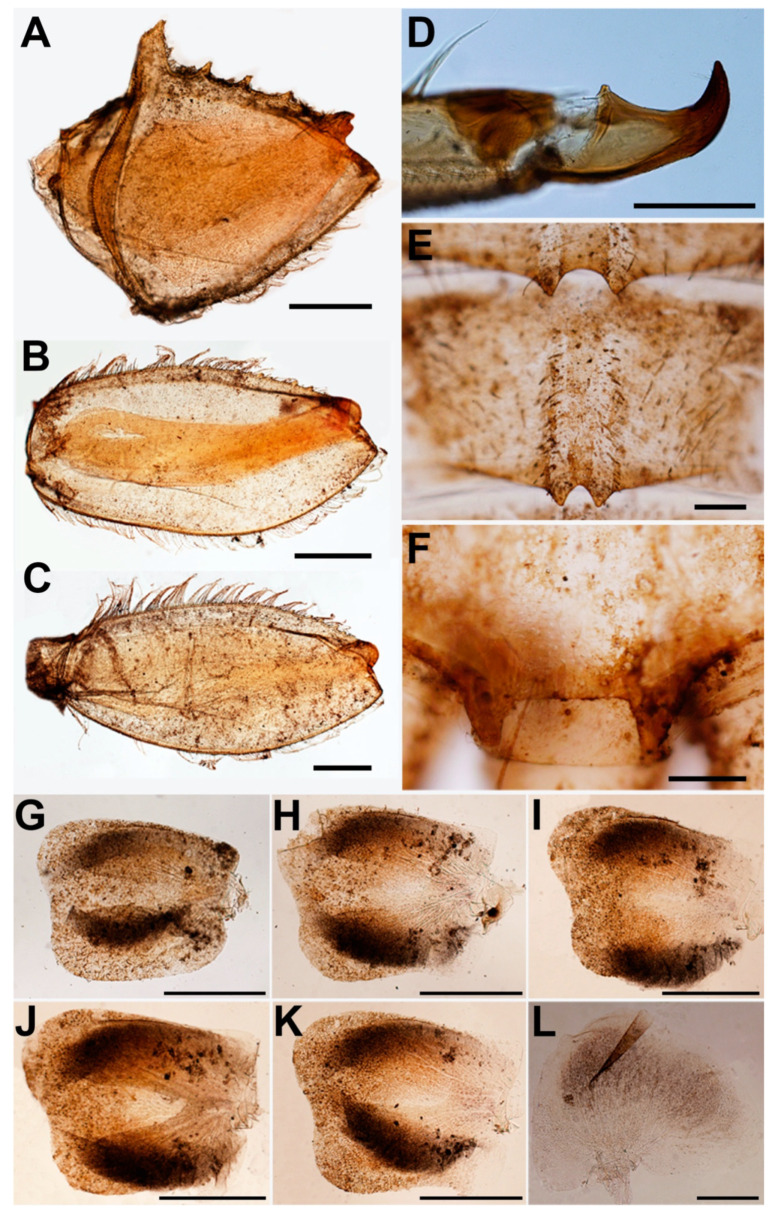
*Vietnamella nanensis* sp. n. (**A**) forefemur; (**B)** midfemur; (**C**) hindfemur; (**D**) foretarsal claw; (**E**) abdominal terga VI–VII; (**F**) abdominal tergum X; (**G**) gill II; (**H**) gill III; (**I**) gill IV; (**J**) gill V; (**K**) gill VI; (**L**) gill VII. Scale bars: 0.5 mm (**A–C**, **E–K**), 0.2 mm (**D**, **L**).

**Figure 4:**
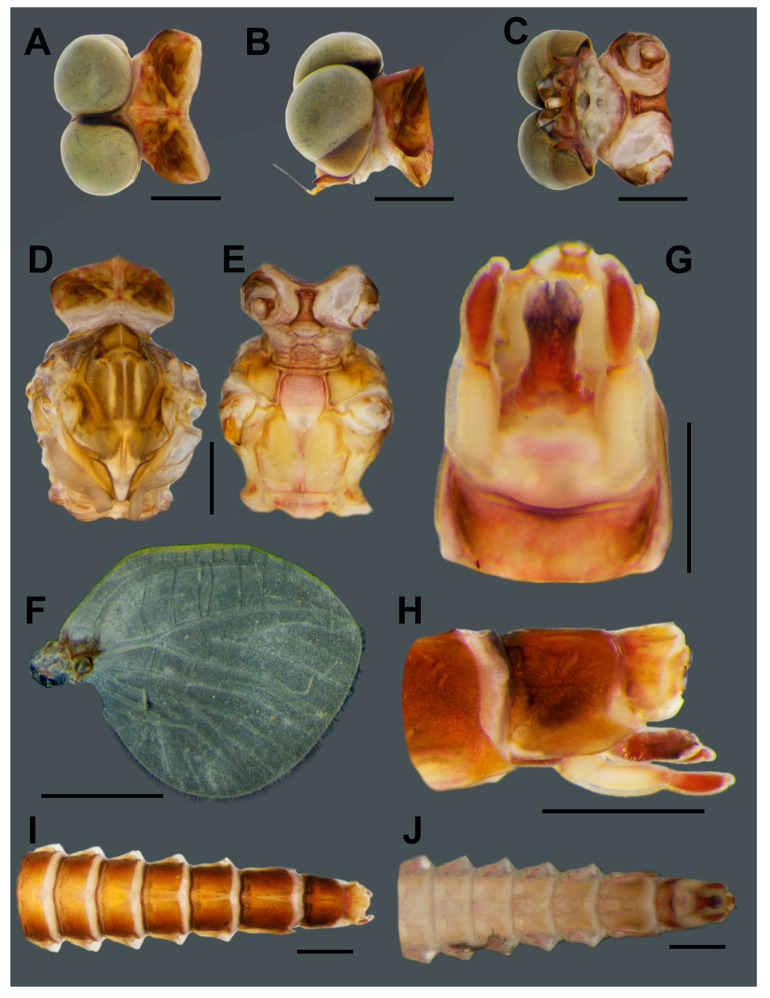
Male subimago of *Vietnamella nanensis* sp. n. Dorsal (**A**), lateral (**B**) and ventral (**C**) views of head; dorsal (**D**) and ventral (**E**) views of thorax; (**F**) hindwing; ventral (**G**) and lateral (**H**) views of genitalia; dorsal (**I**) and ventral (**J**) views of abdomen. Scale bars: 1 mm.

**Figure 5:**
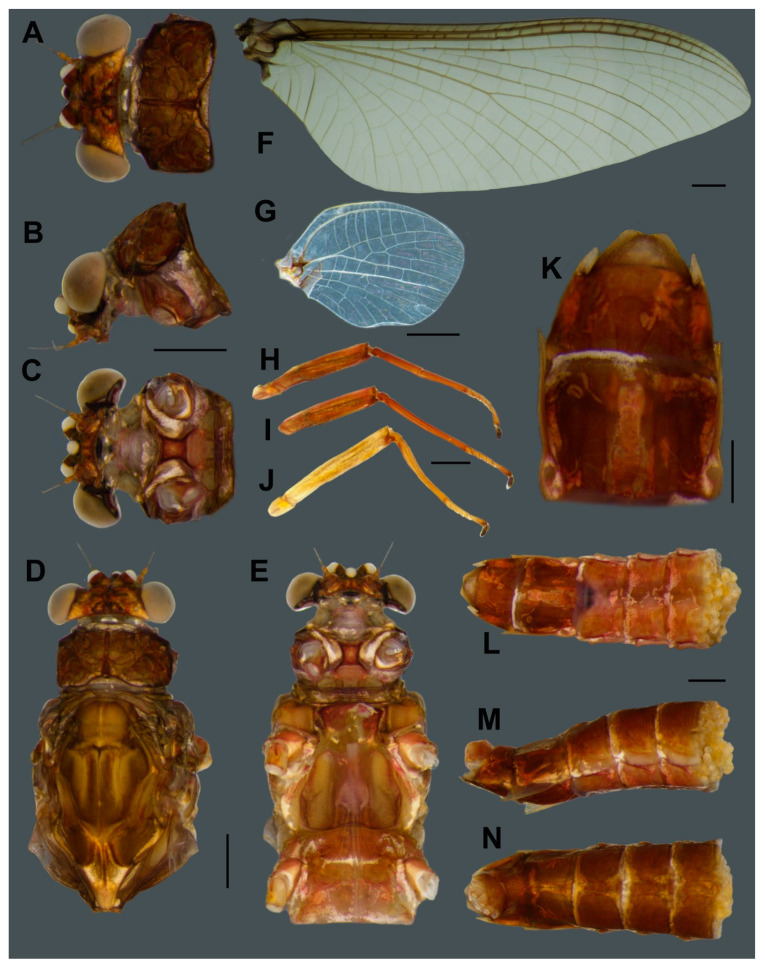
Female imago of *Vietnamella nanensis* sp. n. Dorsal (**A**), lateral (**B**) and ventral (**C**) views of head; dorsal (**D**) and ventral (**E**) views of thorax; (**F**) forewing; (**G**) hindwing; (**H**) foreleg; (**I**) middle leg; (**J**) hindleg; (**K**) ventral view of genitalia; ventral (**L**), lateral (**M**) and dorsal (**N**) views of abdomen. Scale bars: 1 mm.

**Figure 6:**
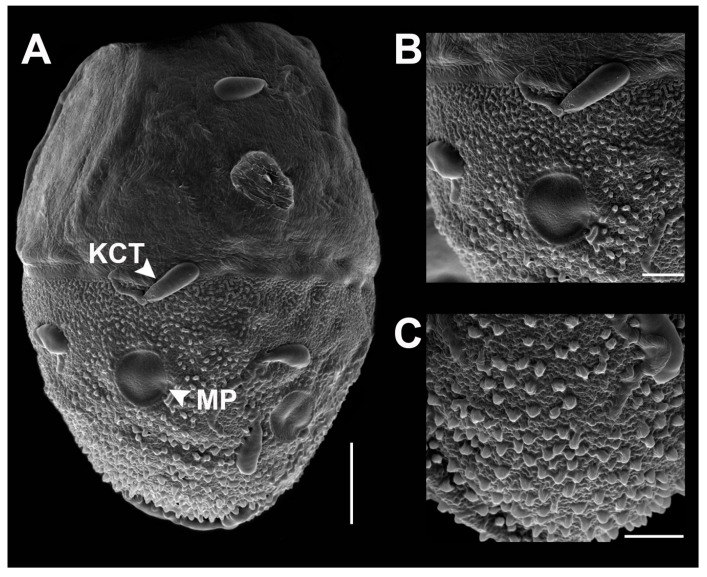
SEM of egg structure of *Vietnamella nanensis* sp. n. (**A**) overview of egg structure; **(B**) detail of Knob terminated Coiled Thread (KCT) and micropyle; (**C**) chorionic surface of posterior area. Scale bars 50 µm (**A**); 20 µm (**B**); 10 µm (**C**).

**Figure 7:**
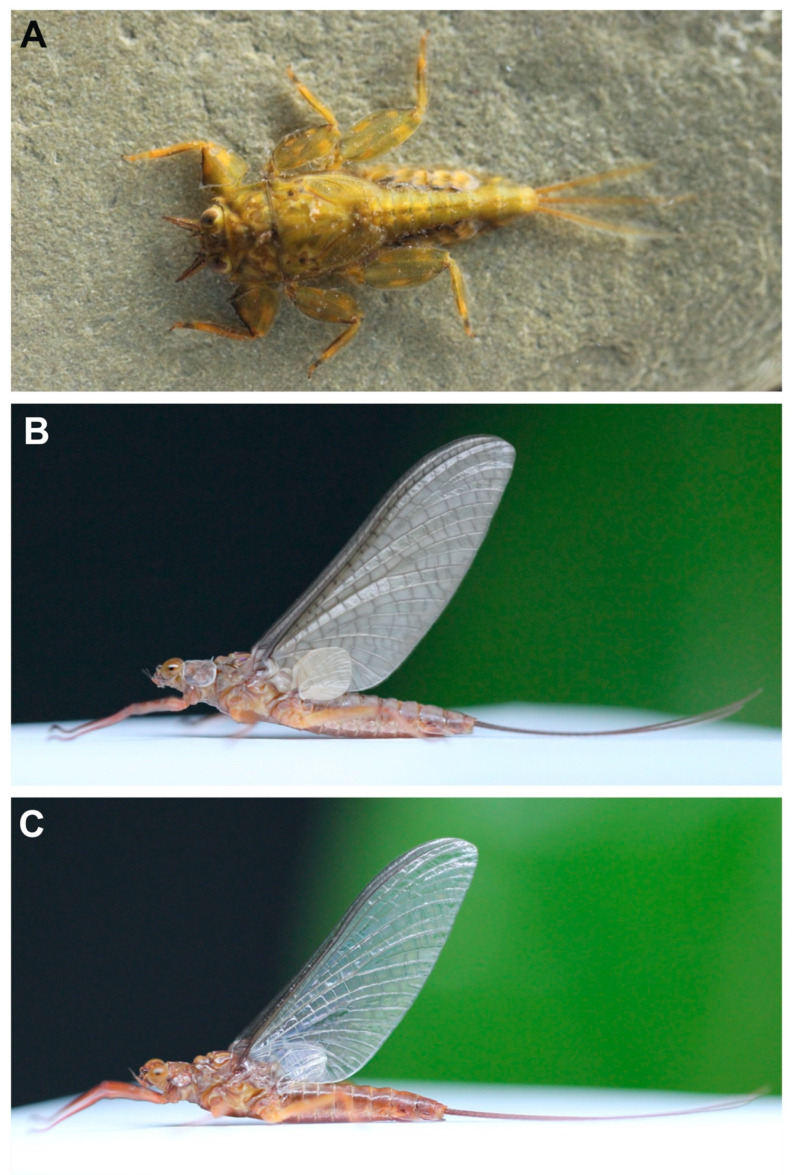
*Vietnamella nanensis* sp. n. (**A**) habitus of larva; (**B**) habitus of female subimago; (**C**) habitus of female imago.

*Vietnamella nanensis* Auychinda & Boonsoong, sp. n. (Figure 1, Figure 2, Figure 3, Figure 4, Figure 5, Figure 6 and Figure 7)

*Vietnamella* sp. C *sensu* Auychinda et al., 2020

**Material examined. *Holotype***: Male mature larva, Thailand, Nan Province, Bo Kluea District, Mae Nam Wa, Wa River, 19°16′22.6” N 101°10′48.2” E, 848 m, 26.XI.2019, B. Boonsoong col. [**ZMKU**]. Paratypes: 24 larvae, 1 male subimago, 2 female subimagoes (incomplete), 2 female imagoes in ethanol, 1 larva on slide, same data as holotype [**ZMKU**]; one larva in ethanol, same data as holotype [**MZL** GBIFCH00834909]. Four larvae, 1 female imago, same locality, but 20.III.2020, B. Boonsoong col. [**ZMKU**]. Additional material: 1 larva, Thailand, Nan Province, Amphur Bo Kluea, Ban Bo Kluea Tai, Nam Mang, 663 m elevation, 19°09.141′ N, 101°09.277′ E, 17.IV.2009, Sites, Vitheepradit, Prommi col. (“L-1044”), BOLD ID: THMAY031-09/Sample ID: 09THMAY-031/Tray ID: L09THMAY-O06 [**PERC**]. Two larvae, Thailand, Mae Hong Son Province, Amphur Khun Yaum, Tumbon Khun Yaum, Huay Ma Surin at Ban Mae Surin, 408 m elevation, 18°54.588′ N, 97°56.695′ E, 20.IV.2009, Sites, Vitheepradit, Prommi col. (“L-1050”), BOLD ID: THMAY148-12/Sample ID: LTHSIT087/Tray ID: BIOUG00646-E12, BOLD ID: THMAY149-12/Sample ID: LTHSIT089/Tray ID: BIOUG00646-F01 [**PERC**].

**Description.**

**Mature larva** (in alcohol, Figure 1; living, Figure 7A). Body length 10–14 mm (*n* = 6) without cerci; cerci 8–10 mm; body brown with pale bandings on thorax and femora (Figure 1A).

**Head.** Brown with pair of occipital tubercles, single sub-occipital tubercle medially; two pairs of projections below eyes; inner pair small, spine-like, and sharp; outer pair large, triangular, cone-shaped, without any serrated spines (Figure 1B). Left mandible slender, outer margin slightly concave at middle; molar block-like shape (Figure 2A). Right mandible slender, outer margin slightly concave at middle; molar block-like shape with tuft of setae below inner molar margin (Figure 2B). Maxillae slender; maxillary palpi three-segmented, with tiny setae, length ratio from basal to apical segments = 1:1.3:1 (Figure 2C,D). Labium: glossae width twice greater than length, glossae with densely short setae anteriorly, outer margins of paraglossae with long setae; labial palpi three-segmented, basal segment broader and longer than second, apical segment small, cone shape; palp with tiny setae most abundant on outer margin (Figure 2E). Labrum: anterior margin with dense short, pectinate setae; anterior half of dorsal surface and margins with relatively long pectinate setae (Figure 2F). Hypopharynx: lingua rounded with anterolateral emargination and superlinguae nearly round, with setae on surfaces (Figure 2G).

**Thorax**. Pronotum with moderately sharp anterolateral projections and slightly pointed protuberances below anterolateral projection. Mesonotum without projections and tubercles. Forefemora strongly expanded with serrations or tooth-like projections on ventral margin (Figure 3A); transverse ridge serrated with spatulate setae and long, thin setae near inner dorsal margin; dorsal and ventral margins with simple, fine setae. Midfemora moderately expanded, dorsal margin smooth and with row of hair-like setae, ventral margin with small serration apically (Figure 3B). Hindfemora moderately expanded, more slender than midfemora, dorsal margin smooth, with row of hair-like setae; ventral margin with small serration apically (Figure 3C). All claws similar, strongly hooked, each with basal protuberance and 4–5 fine sub-apical setae (Figure 3D).

**Abdomen.** Terga covered with simple, short setae; Terga I–X each with pair of median ridges or tubercles; posterolateral angles of terga II–X extended into sharp projections with dense simple setae; terga VI–VII each with pair of acute tubercles (Figure 1C and Figure 3E) and tergum X with well-developed pair of blunt tubercles (Figure 3F). Gills I finger-like with setae; gills on segments II–VI similar in structure (Figure 3G–K); gills on segment VII small, with ventral lamella divided into three lobes (Figure 3L). Caudal filaments with long setae on both lateral side at the middle part.

**Male subimago** (in alcohol, Figure 4). Body length 12 mm without cerci.

**Head.** Eyes with dorsal part yellowish and ventral part pale brown (Figure 4A–C).
**Thorax.** Mesonotum brown with median longitudinal suture (Figure 4D). Mesosternum pale with square basisternum and broad furcasternum (Figure 4E). Hindwing (Figure 4F) with veins and crossveins pale.
**Abdomen.** Genitalia with penis and forceps relatively short and broad; penis with apicomedian emargination, length almost equal to second forceps segment; forceps with total length = 0.96 mm, basal:median segment ratio = 1.4:1 (Figure 4G–H). Terga reddish brown; segments VIII–IX with pair of pale median lines (Figure 4I); sterna light brown; segment IX, penis and forceps with dark brown markings (Figure 4J).

**Female imago** (in alcohol, Figure 5; living, Figure 7C).

**Head.** Eyes pale brown (Figure 5A–C).
**Thorax.** Mesonotum brown with notable median longitudinal suture (Figure 5D). Mesoternum brown with rectangular basisternum and broad furcasternum (Figure 5E). Forewing stigma area without divided longitudinal vein; C to RA area brown; MA forked at middle of wing; MP forked basally, three intercalaries between MP1 and MP2; CuA and CuP adjacent at base (Figure 5F). Hindwing rounded, leading margin slightly concave, with clear cross-veins; 11 cross-veins between Sc and RA, five cross-veins between MA and MP (Figure 5G). Forelegs (6.31 mm) with length ratio of femur:tibia = 1:1.06 (Figure 5H). Midlegs (6.63 mm) with length ratio of femur:tibia = 1:1.3 (Figure 5I). Hindlegs (6.63 mm) with length ratio of femur:tibia = 1:1.4 (Figure 5J).
**Abdomen.** Subanal plate brown with shallow median cleft (Figure 5K). Sterna VIII–IX dark reddish brown; subgenital plate straight, weakly developed, with dark banding (Figure 5L). Segments VIII–IX with prominent spur like projections laterally (Figure 5M). Terga reddish brown (Figure 5N).
**Eggs** (dissected from imago, Figure 6). Ovoid, with length approximately 310 µm, width approximately 220 µm; nearly half of egg covered with helmet-shaped polar cap (Figure 6A). Rod-shaped knob terminated coiled thread (KCT) around egg body; 2 or 3 tagenoform-type micropyles at center, without protuberances in micropyle area (Figure 6B). Chorionic surface with protuberances, prominent in posterior area (Figure 6C).

**Female subimago** (Figure 7B). Body length 12–14 mm without cerci.

**Figure 8:**
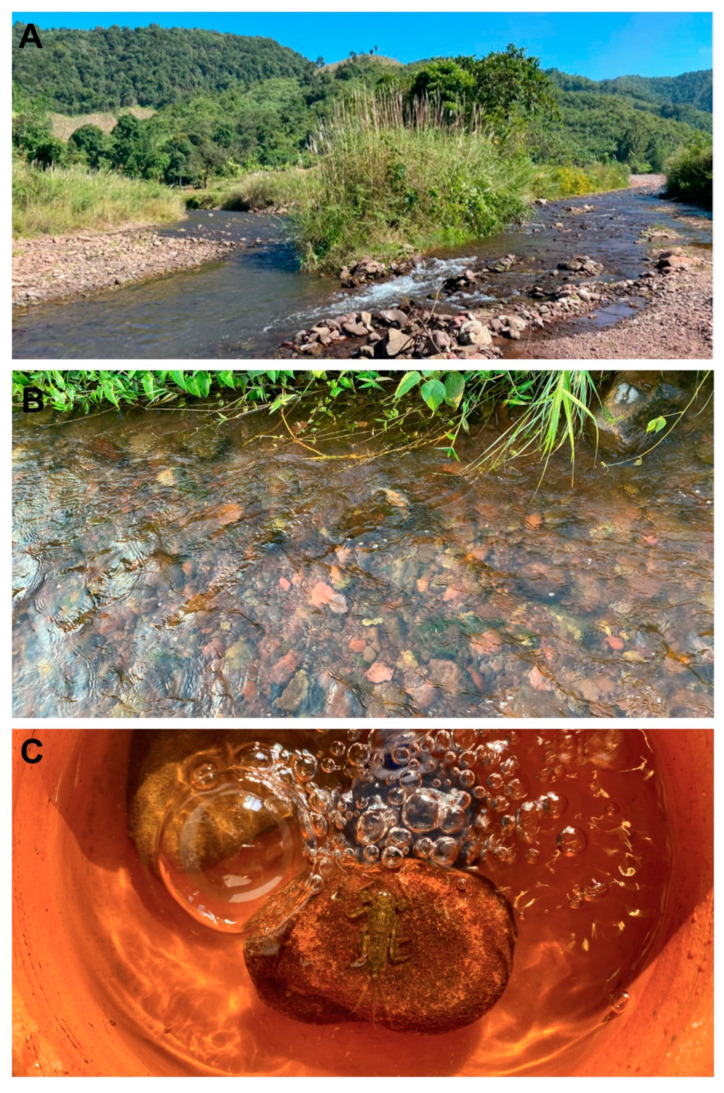
*Vietnamella nanensis* sp. n. (**A**) type locality-Wa river, Nan province, Thailand; (**B**) microhabitat; (**C**) rearing chamber.

**Head.** Eyes yellowish.
**Thorax.** Brownish veins and crossveins brown.
**Abdomen.** Brownish, terga segment VI–IX with pair of median pale line (similar to male subimago).
**Diagnosis.** The larva of *Vietnamella nanensis* sp. n. is most similar to that of *V. thani* Tshernova, but can be separated from the latter and other *Vietnamella* species based on the following combination of characteristics: (i) pattern of serration on the ventral margin of the forefemur (Figure 1A,B and Figure 3A), (ii) posterolateral margins of the abdominal terga with pairs of acute tubercles, especially in terga VI and VII (Figure 1A,C and Figure 3E), (iii) well-developed pair of median ridge projections of tergum X (Figure 3F), (iv) second segment of the maxillary palp being slightly longer than the third segment (1.3:1), and (v) chorionic surface with prominent protuberances (imago stage).
In the alate stages, the anterior margin of forewings is brown and has fewer cross-veins than in the other species. The venation of hind wings shows cross veins between Subcosta (Sc) and Radius sector (RA) similar in number with *V. sinensis* [31] but more numerous compared to *V. thani*. The penis of the subimago is most similar to that of *V. thani* and clearly different from the one of *V. ornata* (Tshernova) [24].
**Remarks.** The morphology of the alate stages was described from male subimago and female imago associated with larvae by rearing method. In addition, the female subimago was photographed before its imago emerged (Figure 7B).
**Etymology.** The specific epithet is a reference to the province Nan in Thailand, where the type material was collected and reared.
**Habitat and ecology.** The type locality of *Vietnamella nanensis* sp. n. is Mae Nam Wa, Nan Province, Thailand (Figure 8A). The larvae were found on cobble and pebbles within the moderate to fast-flowing current of run/riffle areas (Figure 8B). Larvae of *Vietnamella nanensis* sp. n. co-occurred with other Ephemerelloidea larvae, such as *Cincticostella insolta* (Allen), *Notacanthella quadrata* (Kluge & Zhou), *N. commodema* (Allen) (Ephemerellidae), and *Dudgeodes* sp. (Teloganodidae).

**Distribution.** Northern Thailand (Nan and Mae Hon Son provinces).

### 3.2. Identification Key to Known Mature Larvae of Vietnamella Species

1  Head with serration of outer projections··································································································**·········**····2

 Head without serration of outer projections································································**·········**································3

2  Abdominal tergum VII with a pair of tubercles on posterior margin·······························**··*Vietnamella* sp. A**

 Abdominal tergum VII with a single tubercle on posterior margin**·····································*Vietnamella* sp. B**

3  Second segment of maxillary palp greater than half the length of other segments**························*V. sinensis***

 Second segment of maxillary palp nearly equal in length to the other segments**···········································**4

4  Transverse ridge of forefemur with small rounded setae·······························································***V. maculosa***

 Transverse ridge of forefemur with spatulate setae·····························································································5

5  Abdominal tergum VII with a pair of acute tubercles on posterior margin and Posterolateral projection of segment X well developed**······································································································*V. nanensis* sp. n.**

 Abdominal tergum VII with a pair of blunt tubercles on posterior margin and posterolateral projection of segment X less developed**·······················································································································*V. thani***

### 3.3. Molecular Analysis

**Figure 9:**
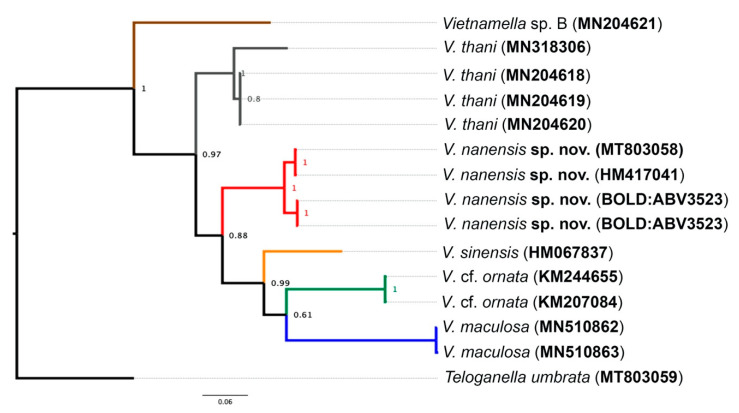
Bayesian interference of Vietnamellidae. The COI phylogenetic reconstruction of six different species of *Vietnamella* with the branch probability support. The accession number of GenBank or Barcode Index Number (BIN) of Barcode of Life Data System (BOLD) in brackets. *Teloganella umbrata* was used as the outgroup.

The COI analysis revealed three major clades, namely *Vietnamella* sp. B, *V. thani*, and a group of *Vietnamella* species with distribution in Northern Thailand and China. The last major clade was distinguished into four clusters and referred to four different species: *V. maculosa* Auychinda, Sartori & Boonsoong, *V.* cf. *ornata*, *V. sinensis*, and *V. nanensis* sp. n. The *V. nanensis* sp. n. cluster is divided into two branches based on geography including Mae Hon Son and Nan provinces (Figure 9).

The intraspecific genetic distances varied between 0% and 3.4%, whereas interspecific distances were very high, ranging from 16.1% to 31.2% (Table 2).

## 4. Discussion and Conclusions

The currently known larvae of *Vietnamella* species can be clearly distinguished from one another by their morphology, and their character comparison is shown in Table 3. *Vietnamella ornata* is not included, as it has been reported only from the subimaginal stage [24]. *Vietnamella* sp. A from India might represent the larva of *V. ornata* [27]; more work is needed in this area. Both morphological and molecular evidence shows similar preliminary results for species numbers and delimitation.

We found that the venation of the stigma area of the forewing can be used to separate *Vietnamella* species into two groups as follows: i) with divided longitudinal vein, including species that have been found only in China (*V. sinensis* and *V. ornata*) and ii) without divided longitudinal vein, including three species (*V. nanensis* sp. n., *V. maculosa* and *V. thani*). The MA fork position of forewings of *V. nanensis* sp. n. shows intraspecific variation, with one specimen having the MA fork situated medially and another having it submedially. The penis of the male subimago of *V. nanensis* sp. n. is most similar to *V. thani,* whereas it differs completely from *V. ornata*. Therefore, the combination of wing and male genitalia characters can be considered for species diagnosis of the adult stages. The details of the adult characters are presented in Table 4.

The chorionic structure can be used for distinguishing between the four species *V. sinensis* [25], *V. maculosa*, *V. thani* [26], and *V. nanensis* sp. n. The eggs of *Vietnamella* show a unique body shape and the helmet-like shape of the polar cap. Differences exist in the details of the chorionic surface and the KCT shape (Table 3). *Vietnamella nanensis* sp. n. shows prominent protuberances on the posterior surface, and the egg size is the largest of the four species. The COI reconstruction showed the existence of six species of *Vietnamella,* in agreement with a previous study [26].

## Figures and Tables

**Table 1:** The mitochondrial cytochrome c oxidase subunit I (COI) sequences for molecular analysis.

Species	Collection Locality	Accession Number/BINs
*Vietnamella nanensis* sp. n.	Mae Hon Son, Thailand	BOLD:ABV3523
	Mae Hon Son, Thailand	BOLD:ABV3523
	Nan, Thailand	HM417041 BOLD:AAG5742
	Nan, Thailand	MT803058
*Vietnamella* cf. *ornata*	Yunnan, China	KM207084.1
	Guangdong, China	KM244655.1
*Vietnamella* sp. B	Tak, Thailand	MN204621
*V. sinensis*	Hubei, China	HM067837.1
*V. maculosa*	Chiang Rai, Thailand	MN510862MN510863
*V. thani*	Prachuap Khiri Khan, Thailand Kanchanaburi, Thailand	MN318306 MN204618 MN204619 MN204620
*Teloganella umbrata*	Narathiwat, Thailand	MT803059

**Table 2:** Pairwise genetic distances (COI) between species of *Vietnamella* using the K2P.

Taxa	K2P Genetic Distances					
	1	2	3	4	5	6
1. *Vietnamella nanensis* sp. n.						
2*. Vietnamella sinensis*	0.187					
3. *Vietnamella maculosa*	0.275	0.258				
*4. Vietnamella* cf. *ornata*	0.245	0.217	0.253			
*5. Vietnamella* sp. B	0.283	0.312	0.289	0.280		
6*. Vietnamella thani*	0.161	0.187	0.256	0.268	0.256	
7. *Teloganella umbrata*	0.275	0.306	0.348	0.373	0.304	0.280

**Table 3:** Comparisons of known larvae and eggs of Vietnamellidae.

Characters	*Vietnamella nanensis* sp. n.	*V. maculosa*	*V. sinensis*	*V. thani*	*Vietnamella* sp. A	*Vietnamella* sp. B
Maxillary palp segment ratio	1:1.3:1	1.3:1.2:1	1:1.6:1	1.3:1.3:1	1:0.9:0.7	1.3:1:1.1
Outer pair of projections on head	Without serration	Without serration	Without serration	Without serration	With serration	With serration
Median ridge projection of abdominal terga	Pair: I–X, well-developed, acute tubercles.	Pair: I–X, moderately developed, blunt tubercles.	Pair: I–X, moderately developed, blunt tubercles.	Pair: I–IX, moderately developed, blunt tubercles.	Pair: II–IX, moderately developed, blunt tubercles.	Pair: II–VI, VIII–X; Single: VII
Posterolateral projection on tergite X	Well developed	Well developed	Moderately developed ^a^	Less developed	Moderately developed ^b^	Moderately developed
KCT shape of eggs	Rod shape	Rod shape	Oval shape	Oval shape	NA	NA
Chorionic surface	Densely notable protuberances	Small protuberances	Small protuberances	Small protuberances	NA	NA
Distribution	Thailand	Thailand	China	Vietnam, Thailand, China	India	Thailand

^a^ [25] Definition based on Figure 1A; p. 383.^b^ [27] Definition based on Figure 1; p. 995.

**Table 4:** Comparison of adult characteristics of known *Vietnamella* species.

Characters	Forewing	Hindwing	Genitalia
*V. nanensis* sp. n. (imago)	Stigma area: not divided by longitudinal vein; 9 cross-veins♀	11 cross-veins between Sc and RA; 5 cross-veins between MA and MP♀	Penis: NA Female subgenital plates: straight
*V. nanensis* sp. n. (subimago)	Stigma area: not divided by longitudinal vein♂	8 cross-veins between Sc and RA; 4 cross-veins between MA and MP♂	Penis: slender, concave medially, deep median cleft Female subgenital plates: NA
*V. ornata* (subimago) ^a^	Stigma area: divided by longitudinal vein♂	15 cross-veins between Sc and R; 7 cross-veins between MA and MP♂	Penis: short and stout, penis tip as long as forceps basal segment Female subgenital plates: NA
*V. sinensis* (imago) ^b^	Stigma area: divided by longitudinal vein♂	12 cross-veins between Sc and RA; 5 cross-veins between MA and MP♂	Penis: slender, shallow median cleft Female subgenital plates: Slightly convex
*V. thani* (imago) ^c^	Stigma area: not divided by longitudinal vein; 17 cross-veins♀	8 or 9 cross-veins between Sc and RA; 3 cross-veins between MA and MP♂ 11 or 12 cross-veins between Sc and RA; 7 cross-veins between MA and MP♀	Penis: slender, shallow median cleft Female subgenital plates: convex
*V. thani* (subimago) ^c^	Stigma area: not divided by longitudinal vein; 16 cross-veins♂	8 or 9 cross-veins between Sc and RA; 6 cross-veins between MA and MP♂	Penis: slender, shallow median cleft Female subgenital plates: NA

^a^ [24] Based on Figure 6; p. 613.^b^ [25] Based on Figure 4A,C and Figure 5A,C; p. 385. ^c^ [26] Based on Figure 8H,J; p. 29.

## References

[1] Soldán T., Landolt P., Sartori M. (1997). Mayflies (Ephemeroptera): One of the earliest insect groups known to man. Ephemeroptera & Plecoptera Biology-Ecology-Systematics.

[2] Sartori M., Brittain J.E., Thorp J.H., Rogers D.C. (2015). Order Ephemeroptera. Ecology and General Biology, Vol I: Thorp and Covich’s Freshwater Invertebrates.

[3] Barber-James H.M., Gattolliat J.-L., Sartori M., Hubbard M.D. (2008). Global diversity of mayflies (Ephemeroptera, Insecta) in freshwater. Hydrobiologia.

[4] Macadam C.R., Stockan J.A. (2015). More than just fish food: Ecosystem services provided by freshwater insects. Ecol. Entomol..

[5] Jacobus L.M., Macadam C.R., Sartori M. (2019). Mayflies (Ephemeroptera) and their contributions to ecosystem services. Insects.

[6] Beattie A., Ehrlich P.R. (2001). Wild Solutions: How Biodiversity Is Money in the Bank.

[7] Boonsoong B., Sangpradub N., Barbour M.T. (2009). Development of rapid bioassessment approaches using benthic macroinvertebrates for Thai streams. Environ. Monit. Assess..

[8] Arimoro F.O., Muller W.J. (2010). Mayfly (Insecta: Ephemeroptera) community structure as an indicator of the ecological status of a stream in the Niger Delta area of Nigeria. Environ. Monit. Assess..

[9] Stepanian P.M., Entrekin S.A., Wainwright C.E., Mirkovic D., Tank J.L., Kelly F. (2020). Declines in an abundant aquatic insect, the burrowing mayfly, across major North American waterways. PNAS..

[10] Kluge N.J., Novikova E.A. (2017). Occurrence of *Anafroptilum* Kluge 2012 (Ephemeroptera: Baetidae) in Oriental Region. Zootaxa.

[11] Sutthinun C., Gattolliat J.-L., Boonsoong B. (2018). A new species of *Platybaetis* Müller-Liebenau, 1980 (Ephemeroptera: Baetidae) from Thailand, with description of the imago of *Platybaetis bishopi* Müller-Liebenau, 1980. Zootaxa.

[12] Kluge N.J., Suttinun C. (2020). Review of the Oriental genus *Indocloeon* Müller-Liebenau 1982 (Ephemeroptera: Baetidae) with descriptions of two new species. Zootaxa.

[13] Auychinda C., Sartori M., Boonsoong B. (2020). Review of *Notacanthella* Jacobus & McCafferty, 2008 (Ephemeroptera: Ephemerellidae) in Thailand, with the redescription of *Notacanthella commodema* (Allen, 1971). Zootaxa.

[14] Boonsoong B., Braasch D. (2013). Heptageniidae (Insecta, Ephemeroptera) of Thailand. ZooKeys.

[15] Boonsoong B., Sartori M. (2015). A new species of *Compsoneuriella* Ulmer, 1939 (Ephemeroptera: Heptageniidae) from Thailand. Zootaxa.

[16] Sutthacharoenthad W., Sartori M., Boonsoong B. (2019). Integrative taxonomy of *Thalerosphyrus* Eaton, 1881 (Ephemeroptera, Heptageniidae) in Thailand. J. Nat. Hist..

[17] Boonsoong B., Sartori M. (2015). The nymph of *Gilliesia* Peters & Edmunds, 1970 (Ephemeroptera: Leptophlebiidae), with description of a new species from Thailand. Zootaxa.

[18] Boonsoong B., Sartori B. (2016). *Sangpradubina*, An astonishing new mayfly genus from Thailand (Ephemeroptera: Leptophlebiidae: Atalophlebiinae). Zootaxa.

[19] Boonsoong B., Sartori M. (2019). Review and integrative taxonomy of the genus *Prosopistoma* Latreille, 1833 (Ephemeroptera, Prosopistomatidae) in Thailand, with description of a new species. ZooKeys.

[20] Martynov A.V., Palatov D.M., Boonsoong B. (2016). A new species of *Dudgeodes* Sartori, 2008 (Ephemeroptera: Teloganodidae) from Thailand. Zootaxa.

[21] Piraonapicha K., Sangpradub N. (2019). Description of nymphs and female subimago of *Sparsorythus multilabeculatus* Sroka & Soldán, 2008 (Ephemeroptera: Tricorythidae) associated with male imago based on DNA sequence data. Zootaxa.

[22] Jacobus L.M., McCafferty W.P. (2006). Reevaluation of the phylogeny of the Ephemeroptera infraorder Pannota (Furcatergalia), with adjustments to higher classification. Trans. Am. Entomol. Soc..

[23] Ogden T.H., Breinholt J.W., Bybee S.M., Miller D.B., Sartori M., Shiozawa D., Whiting M.F. (2019). Mayfly phylogenomics: Initial evaluation of anchored hybrid enrichment data for the order Ephemeroptera. Zoosymposia.

[24] Tshernova O.A. (1972). Some new species of mayflies from Asia (Ephemeroptera, Heptageniidae, Ephemerellidae) (in Russian). Rev. Entomol. URSS.

[25] Hu Z., Ma Z.X., Luo J.Y., Zhou C.F. (2017). Redescription and commentary on the Chinese mayfly *Vietnamella sinensis* (Ephemeroptera: Vietnamellidae). Zootaxa.

[26] Auychinda C., Sartori M., Boonsoong B. (2020). Vietnamellidae (Insecta, Ephemeroptera) of Thailand. ZooKeys.

[27] Selvakumar C., Sinha B., Vasanth M., Subramanian K.A., Sivaramakrishnan K.G. (2018). A new record of monogeneric family Vietnamellidae (Insecta: Ephemeroptera) from India. J. Asia-Pac. Entomol..

[28] Hwang J.M., Seateun S., Nammanivong M., Bae Y.J. (2010). Ephemeroptera fauna of Nam Et National Biodiversity Conservation Area in Laos. Bull. Entomol. Res..

[29] Jacobus L.M., McCafferty W.P., Sites R.W. (2005). Significant range extensions for *Kangella* and *Vietnamella* (Ephemeroptera: Ephemerellidae, Vietnamellidae). Entomol. News..

[30] Folmer O., Black M., Hoeh W., Lutz R., Vrijenhoek R. (1994). DNA primers for amplification of mitochondrial cytochrome c oxidase subunit I from diverse metazoan invertebrates. Mol. Mar. Biol. Biotechnol..

[31] Hsu Y.C. (1936). New Chinese mayflies from Kiangsi Province (Ephemeroptera). Peking Nat. Hist. Bull..

